# Fatal temozolomide induced aplastic anemia in a female with Glioblastoma multiforme : A case report and literature review

**DOI:** 10.1002/ccr3.3860

**Published:** 2021-01-28

**Authors:** Fateen Ata, Safna Farsana Akkam Veettil, Mohammed Gaber, Nabil E. Omar, Ammar Madani, Hebatalla Mah. Afifi, Meeloud M. Aldardouri, Aliaa Amer, Samah Kohla, Abdul Rehman Zar Gul

**Affiliations:** ^1^ Department of Internal Medicine Hamad General Hospital Hamad Medical Corporation Doha Qatar; ^2^ Department of Oncology National Center for Cancer Care & Research Hamad Medical Corporation Doha Qatar; ^3^ Pharmacy Department National Center for Cancer Care & Research Hamad Medical Corporation Doha Qatar; ^4^ Hematopathology Division, Department of Laboratory Medicine and Pathology Hamad Medical Corporation Doha Qatar

**Keywords:** aplastic anemia, glioblastoma multiforme, temozolomide

## Abstract

When seeing patients on Temozolomide with pancytopenia, aplastic anemia secondary to the drug should be considered early in the differentials to avoid permanent hematological suppression.

## Graphical abstract

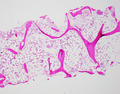

## 1 INTRODUCTION

Temozolomide (TMZ) is a standard of care treatment for Glioblastoma Multiforme (GBM). We report a fatal outcome of a 55‐years‐old female with GBM who developed persistent aplastic anemia (AA) while being treated with TMZ. This case highlights the possibility of a dose‐independent and fatal AA secondary to TMZ.

Temozolomide, an alkylating agent, is a chemotherapeutic drug used mainly in GBM.^1^ It was approved for use by the Food and Drug Administration (FDA) in 2005.^2^ Since then, it is being used globally in patients with GBM to reduce disease progression. TMZ is used initially with radiotherapy for six weeks (concurrent chemo‐radiotherapy‐CRT) and subsequently alone as maintenance chemotherapy. The first maintenance therapy cycle typically starts 28 days after chemo‐radiotherapy (CRT). The first cycle dose is 150 mg/m2 and 200 mg/m^2^ for subsequent cycles.^3^ Like any chemotherapy agent, TMZ is associated with multiple side effects, including hematological toxicity.^1^ Aplastic anemia is an emerging adverse effect of TMZ.^2^, ^4^, ^5^, ^6^, ^7^, ^8^, ^9^, ^10^, ^11^ The crucial observation about AA secondary to TMZ is its irreversible and dose‐independent nature.^2^, ^4^, ^5^, ^6^, ^7^, ^8^ We have summarized the cases reported in the literature and analyzed them for the patients' age and sex, time of diagnosis of AA after initiation of TMZ therapy, and the nature of AA. We have also reported a case of AA secondary to Temozolamide with characteristics in keeping with most reported cases.

## 2 CASE REPORT

A 55‐year‐old Tunisian female presented to the hospital with confusion, aphasia, and a Glasgow Coma Scale (GCS) of 12/15. A Computed Tomography (CT) scan head revealed two well‐defined adjacent lobulated intra‐axial lesions in the left occipital lobe. MRI head detected multiple left cerebral lesions in the temporo‐occipital area. A stereotactic biopsy of one of the lesions was performed, and histopathology revealed Anaplastic Astrocytoma World Health Organization (WHO) grade 3. The tumor cells were strongly positive with Glial Fibrillary Acidic Protein (GFAP), Oligodendroglial Lineage Marker (OLIG‐2), while negative with CD20 and CD30. Few scattered lymphocytes were seen within the tumor cells staining with CD3, CD8, CD45, and CD4. Ki‐67 index was high, reaching 50% in some areas. Isocitrate Dehydrogenase (IDH) 1 and 2, P53, and BRAF mutations were negative, whereas the ATRX nuclear staining was retained. The patient underwent excision of the tumor after two weeks. Histopathology results of excision biopsy revealed Glioblastoma, WHO grade 4, with wild type IDH‐1, P‐52, ATRX, and a 50% proliferative index of Ki‐67. Post‐operation, she had two episodes of generalized tonic‐clonic seizures and was started on levetiracetam 500 mg twice daily. She also developed pulmonary embolism, for which she was started on enoxaparin twice daily.

One–month post‐surgery, she was started on partial brain radiotherapy and concurrent chemotherapy with Temozolomide at a dose of 75mg/m^2^ for six weeks with the plan to maintain on Temozolomide 150mg/m^2^ alone afterward.

During concurrent ‐chemo‐radiotherapy, after 19 sessions, patient presented with bleeding from injection sites on her thigh and bruises on her upper and lower limbs for one day. She did not complain of headache, fever, or bleeding from any other site. The patient was on enoxaparin and levetiracetam at the time of admission with bleeding and bruises from injection sites. Enoxaparin was withheld due to the bleeding. On examination, she was vitally stable, and apart from limb bruises and oozing blood from the injection site, her physical examination was unremarkable. Blood profile revealed pancytopenia with a white blood cell (WBC) count of 0.2 x10^3/uL (normal range: 4‐10 x10^3/uL), hemoglobin of 8.5 gm/dL (normal range: 12‐15 gm/dL), platelet count of 7 x 10^3^/μL (normal range: 150‐400 × 10^3^/μL) and absolute neutrophil count of 0.0 × 10^3^/μL (normal range: 2‐7 × 10^3^/μL).

Temozolomide was discontinued as it was suspected to be the underlying cause of her pancytopenia. Her absolute reticulocyte count was 0.2 (normal level: > 2), indicating a hypo‐proliferative marrow. Peripheral smear showed marked pancytopenia, but no abnormal cells were visualized. She was initiated on Filgrastim 300 mcg subcutaneous daily and received multiple platelet infusions without any ANC or platelet count improvement. The patient developed febrile neutropenia three days after admission, secondary to the right axillary cellulitis. Swab culture grew Pseudomonas Aeruginosa, for which intravenous cefepime was given for ten days. The patient became afebrile after completion of cefepime but remained pan cytopenic despite platelet and Filgrastim transfusions. Radiotherapy sessions were also held since this admission due to critically low platelet and WBC counts.

**FIGURE 1:**
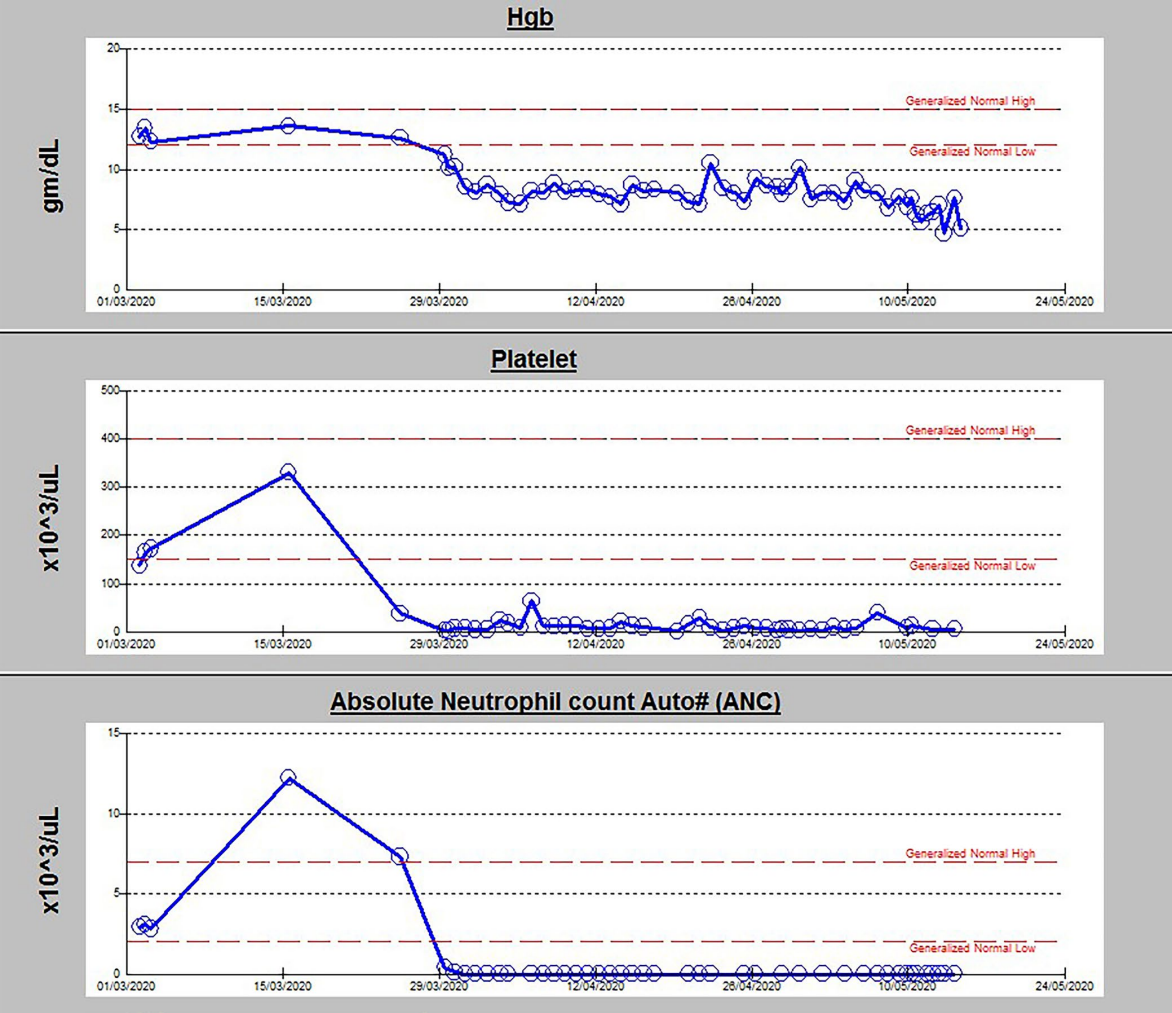
Section of bone marrow trephine biopsy showing marked hypocellularity. Many of the remaining cells are plasma cells. H & E, 4x

Given the non‐resolving pancytopenia without an apparent cause, a bone marrow biopsy was performed. Bone marrow aspirate showed few tiny particles with hypocellular smear and markedly decreased trilineage hematopoiesis, many scattered lymphocytes, and relatively increased plasma cells (28%) with no increase in blasts. Bone marrow biopsy showed pronounced hypocellularity (5%‐15%) with a remarkably decreased trilineage hematopoiesis and a relative increase in plasma cells (Figure 1). Flow cytometry on bone marrow aspirate showed approximately 50% T‐cells, 3% B‐cells, and increased plasma cells (15%) with no immunophenotypic evidence of monotypic B‐cell or plasma cell populations. The bone marrow did not show any evidence of paroxysmal nocturnal hemoglobinuria in flow cytometry analysis. As the patient took Temozolomide, which was recently started, she was diagnosed with aplastic anemia secondary to it.

**FIGURE 2:**
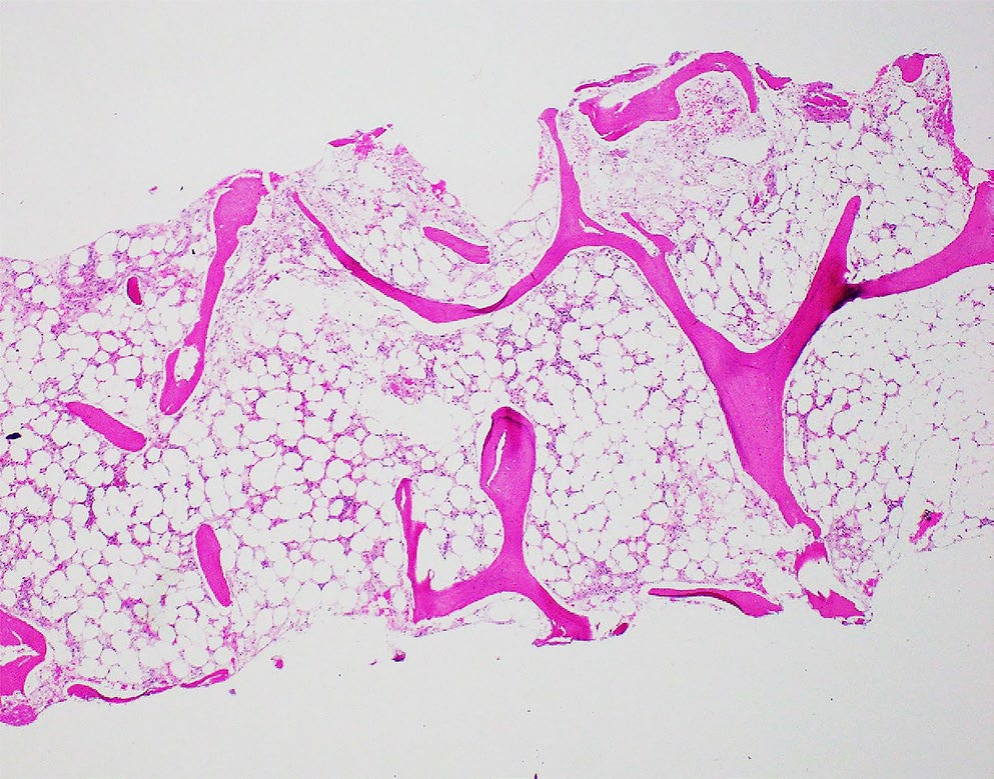
Patient's Hemoglobin (Hgb), Platelet count, and absolute neutrophil count (ANC) during the hospital stay

Two days after the bone marrow biopsy, the patient developed left facial swelling with fever. A CT scan of the face revealed a subtotal opacification of the anterior left nasal cavity with low soft tissue density, extending to the left cheek medially. There was also a partial opacification of both maxillary sinuses, noted more on the left side, with hyperdense foci. The collective picture was suggestive of suspected fungal infection. She was started on IV Meropenem 1 gm 8 hourly and Amphotericin‐B 435 mg daily, empirically, and cultures were sent to the lab. However, the patient remained febrile and pan cytopenic (Figure 2). Treatment options for AA were discussed with the hematology team; however, she was not a candidate for Anti‐thymocyte globulin (ATG) or cyclosporin or because of underlying malignancy and pulmonary embolism. A trial of IV Immunoglobulin was given with no improvement in counts.

The cheek swelling worsened involving the left eye, and an urgent debridement was performed. The infected area's biopsy revealed necrotic tissue, heavily infiltrated by fungal organisms, compatible with aspergillosis. Although the necrotic tissue was debrided, and she was on appropriate antifungal medication, the facial cellulitis did not respond to the treatment, and she deteriorated with desaturation, hypotension, and confusion. On the 38th‐day post‐admission, the patient was transferred to the Medical Intensive Care Unit (MICU) and was intubated to protect the airways and started on vasopressors for her septic shock. The patient remained transfusion‐dependent and was sedated and intubated for a total of 10 days. The patient eventually developed a cardiac arrest with asystole and succumbed to sepsis and pancytopenia with a complicated and prolonged hospital stay of 47 days. Throughout her hospital stay, the patient had bacteremia with Multi‐Drug Resistant Organisms (MDRO) including Stenotrophomonas, Enterococcus casseliflavus, and Achromobacter xylosoxidans, and fungemia with Aspergillus flavus.

## 3 DISCUSSION

GBM is one of the most commonly occurring Central Nervous System (CNS) malignancies, with a prevalence of 5/100 000 people per annum.^12^ Most of the patients are diagnosed between the ages of 75 to 84 years. The prognosis is usually unfavorable, with a five‐year survival rate of less than 5 percent.^12^ Most frequent presentations include headache, seizures, and focal neurological signs, including but not limited to; memory problems, vision changes, motor deficits, and personality changes.^13^ With advancements in radiology and the availability of Magnetic Resonance Imaging (MRI), the diagnosis of GBM is not challenging anymore. The tumor can be seen as a hypodense lesion on T1‐weighted cuts and increased signal intensity post‐contrast.^14^ Biopsy with histopathologic and genetic analysis can confirm the stage and type (primary or secondary).^12^

Three primary modalities of treatment in GBM include surgery, chemotherapy, and a combination of chemo‐radiotherapy. Post‐surgery radiation was the standard treatment method until 2005; however, currently, Temozolomide with radiation therapy is the gold standard management modality, especially in younger patients.^15^ Other treatments include Bevacizumab, immune therapy (checkpoint inhibitors, peptide vaccinations, adoptive cell therapy, viral immunotherapy, and dendritic cell vaccinations).^15^

The landmark EORTC‐NCIC trial paved the way for the use of combination chemo‐radiotherapy with Temozolomide in GBM.^16^ Temozolomide is an alkylating agent, given at a dose of 75 mg/m^2^ with radiation. Subsequent doses are 150 and 200 mg/m^2^ for the first post‐radiation cycle and subsequent cycles, respectively.^1^ Unlike other alkylating agents, one of the rare adverse effects of Temozolomide is aplastic anemia.^2^, ^17^ Similar irreversible AA was observed in our patient, where discontinuation of the drug did not resolve or improve AA.

The cases of AA secondary to Temozolomide are tabulated (Table 1). Most of the patients reported having AA are females (9/10). Other studies have also shown a slight female predominance for hematological complications of Temozolomide.^5^ The median age of diagnosis of AA is 51 years. Most of the patients developed AA during CRT or after CRT and before initiation of TMZ monotherapy. Another noteworthy observation is AA's irreversible nature, with only 3 out of 10 patients recovered or improved to some extent by discontinuation of TMZ, supportive transfusions, and Filgrastim. AA was observed at doses ranging from 75‐200 mg/m^2^, indicating a possible dose‐independent effect.^2^, ^9^ Although the possibility of the contribution of RT in the development of AA cannot be ruled out, incidences of AA during monotherapy and varying doses of TMZ when with RT provide evidence of its hematotoxicity. Another point to consider is the well‐established pattern of AA secondary to RT, which is dose and time‐dependent, unlike the pattern observed in the discussed cases.^18^ A similar approach was adopted by us while evaluating the cause of AA in our patients.

**TABLE 1:** Reported cases of Temozolomide induced Aplastic Anemia

Author, year	Sex, Age	Time of diagnosis of AA	AA during CRT or Monotherapy	Dose of TMZ/day	Reversible	Outcome of AA
Villano et al 2006	M, 45	Fourth Cycle	Monotherapy	200 mg/m^2^	No	Death
George BJ et al 2009	F, 65	Day 14 of CRT	CRT	NA	No	Death
Comez G et al 2010	F, 31	Third Cycle	CRT	150 mg/m^2^	No	Death
Jalali R et al 2007	F, 30	After CRT	CRT	75 mg/m^2^	No	Death
Kopecky J et al 2010	F, 61	Day 23 of CRT	CRT	75 mg/m^2^	No	Death
Morris EB et al 2009	F, 16	Day 24 of CRT	CRT	90 mg/m^2^	No	Death
Nagane M et al 2009	F, 51	Day 20 of CRT	CRT	75 mg/m^2^	Yes	Recovered
Newton SL et al 2018	F, 51	Day 54 from start of TMZ	Monotherapy	150 mg/m^2^	Yes	Improved
Oh J et al 2010	F, 63	Day 18 of CRT	CRT	NA	Yes	Improved
Our patient	F, 55	Fourth Cycle	CRT	75 mg/m^2^	No	Death

Abbreviations: AA, Aplastic Anemia; CRT, chemo‐radiotherapy; F, Female; M, Male; NA, Not available; TMZ, Temozolomide.

The exact mechanism of AA secondary to TMZ is yet to be completely understood and is thought to be immune‐mediated.^5^ However, to somewhat understand the pathophysiology behind the induction of AA secondary to TMZ, it is vital first to appreciate the pharmacodynamics of TMZ on molecular levels. TMZ is innately in its inactive form, and once in the body, it is converted to its active metabolite, MTIC [(methyl‐triazene‐1‐yl)‐imidazole‐4‐carboxamide], spontaneously.^10^ MTIC "methylates the N^7^ and O^6^ position of guanine and the O^3^ position of adenine". Subsequently, the cells' auto‐destruction occurs in an unsuccessful attempt to repair the methylations by the mismatch repair genes.^5^ One such mismatch repair gene, methylguanine‐DNA methyltransferase (MGMT), reverses the methylation at the guanine position and, as a result, the effect of TMZ gets inactivated. Cells that are, by default, deficient in MGMT are more susceptible to TMZ cytotoxicity, which is also the case with hematopoietic precursor cells.^10^

Although not a malignant hematological disease, aplastic anemia can have a high mortality rate if left untreated. The most common causes of AA include cytotoxic drugs and radiation therapy. However, usually, their effect is dose‐dependent and hence, reversible.^19^ Other uncommon causes of AA include toxic chemicals such as Benzene and certain pesticides and viruses such as hepatitis and human immunodeficiency virus.^20^, ^21^ The diagnosis depends on a careful history, focusing on medication history, past medical conditions, and occupation. Relevant tests must rule out other causes of bone marrow suppression before confirming AA. Diagnosis can be confirmed by bone marrow biopsy, which reveals a severely hypocellular marrow in the absence of malignant cells or fibrosis.^22^

The treatment of AA depends much on host factors as well as the degree of AA. The usual approach for patients younger than 40 years of age is hematopoietic cell transplantation (HCT). For older patients, combined immunosuppressive therapy with Anti‐thymocyte globulin, Cyclosporine, and Eltrombopag, is the first‐line modality of treatment, given that the patient is stable enough to tolerate the effects of the combination therapy. For medically unfit patients, symptomatic management is the current standard of care.^23^ Although treatment can be curative, lethal complications can occur, making it a reasonably challenging disease to tackle.^24^

## 4 CONCLUSION

This report aims to highlight the possibility of a dose‐independent and fatal aplastic anemia secondary to Temozolomide. When seeing patients on Temozolomide with pancytopenia, aplastic anemia secondary to the drug should be considered early in the differentials to avoid permanent hematological suppression.

## CONFLICT OF INTEREST

None declared.

## AUTHOR CONTRIBUTIONS

FA‐ contributed to the literature review and drafted the initial manuscript, revisions in the manuscript. SFA‐ designed the idea initially, helped in manuscript writing, critically revised the paper, and proof‐reading the manuscript. MG‐ contributed to the literature review and drafted the initial manuscript. NEO‐ conceived and designed the idea, literature review, data collection, wrote the manuscript, overall organized the case report, supervised the project, and proof‐reading of the manuscript. AM‐ contributed to data collection and helped in manuscript writing. HMA‐ contributed to data collection and helped in manuscript writing. MMA‐ contributed to data collection and helped in manuscript writing. AA‐contributed to the pathology section of the case report. SK‐contributed to the pathology section of the case report. ARZ‐managed patient care and revised the final draft of the manuscript. All authors gave final approval of the version to be published and agreed to be accountable for all aspects of the work.

## ETHICAL APPROVAL

The case report was approved by the Medical Research Centre at Hamad Medical Corporation, Qatar, and the Hamad Institutional Review Board (IRB) under number MRC‐04‐20‐835.

## DECLARATION

This manuscript is original work and has not been submitted or is not under consideration for publication elsewhere. All the authors have reviewed the manuscript and approved it before submission. Name of Department and Institution where work was done: Department of Oncology, National Center for Cancer Care & Research, Hamad Medical Corporation, Doha, Qatar.

## CONSENT FOR PUBLICATION

A written informed consent of patient information, images and publication was signed by the next of kin of patient before the submission of the manuscript.

## Data Availability

The datasets used and/or analyzed during the current study are available from the corresponding author on reasonable request.
